# Medical Appointment

**Published:** 1850-03

**Authors:** 


*Medical Appointment.*—Dr. N. D. Benedict, of the Blockley Hospital, Philadelphia, has been appointed medical Superintendent of the State Lunatic Asylum at Utica, in the place of the late Dr. Brigham.

